# Genesis of Open States Zones in a DNA Molecule Depends on the Localization and Value of the Torque

**DOI:** 10.3390/ijms23084428

**Published:** 2022-04-17

**Authors:** Stepan Dzhimak, Alexandr Svidlov, Anna Elkina, Eugeny Gerasimenko, Mikhail Baryshev, Mikhail Drobotenko

**Affiliations:** 1 Department of Radiophysics and Nanothechnology, Physics Faculty, Kuban State University, 350040 Krasnodar, Russia; svidlov@mail.ru (A.S.); anna013194@mail.ru (A.E.); baryshev_mg@mail.ru (M.B.); mdrobotenko@mail.ru (M.D.); 2 Laboratory of Problems of Stable Isotope Spreading in Living Systems, Federal Research Center the Southern Scientific Center of the Russian Academy of Sciences, 344006 Rostov-on-Don, Russia; 3 Department of Technology of Fats, Cosmetics, Commodity Science, Processes and Devices, Kuban State Technological University, 350072 Krasnodar, Russia; rosmaplus@gmail.com

**Keywords:** DNA dynamics, open states, torque, mathematical model, rotational movements of nitrogenous bases

## Abstract

The formation and dynamics of the open states in a double-stranded DNA molecule are largely determined by its mechanical parameters. The main one is the torque. However, the experimental study of DNA dynamics and the occurrence of open states is limited by the spatial resolution of available biophysical instruments. Therefore, in this work, on the basis of a mechanical mathematical model of DNA, calculations of the torque effect on the process of occurrence and dynamics of open states were carried out for the interferon alpha 17 gene. It was shown that torsion action leads to the occurrence of rotational movements of nitrogenous bases. This influence is nonlinear, and an increase in the amplitude of the torsion action does not lead to an automatic increase in the amplitude of rotational movements and an increase in the zones’ open states. Calculations with a constant torsion moment demonstrate that open states zones are more often formed at the boundaries of the gen and in regions with a predominance of *A–T* pairs. It is shown, that for the occurrence of open states in the part of the gene that contains a small number of *A–T* pairs, a large amount of torque is required. When the torque is applied to a certain region of the gene, the probability of the formation of the open state depends on the content of *A–T* pairs in this region, the size of this region, and on the exposure time. For this mathematical model, open states zones can be closed when the torsion action stops. The simulation results showed that the values of the torsion moment required for the appearance of open states zones, in some cases, are close to experimentally measured (13–15 pN·nm).

## 1. Introduction

Open states (OS) are one of the factors affecting a number of biochemical processes, and play an important role in the processes of transcription and replication, as well as in DNA-protein recognition during the repair of nucleic acids [1,2,3,4,5]. The process of formation and dynamics of OS zones of a double-stranded DNA molecule is largely determined by its mechanical parameters, the most important of which is the torque [6,7,8]. It was noted that DNA torque can play an important role in the process of transcription [9,10,11]. In this regard, the importance of single-molecule level studies has significantly increased, and experimental studies are rapidly developing to measure the torque in DNA molecules [12,13,14]. Thus, using an angled optical trap and magnetic tweezers, the magnitude of the torque leading to the unwinding of the DNA double helix was measured [15,16].

In addition, the amount of torque required to melt the DNA was determined from the difference between the work done in stretching one wound molecule and the work done in stretching one overwound molecule on the same number of turns. [17,18]. It was also found that RNA polymerase is able to generate a torque that is sufficient to change the DNA structure [19]. However, the experimental study of DNA dynamics and OS occurrence is limited by the spatial resolution of the available biophysical tools [20,21]. A more complete description of the DNA mechanics can be obtained by combining data on its mechanical parameters and studying its movements. Such an approach is possible within the framework of mathematical modeling [22,23,24].

In this paper, the dynamics of OS occurrence under the torque influence with different spatial and temporal localization are studied. The processes described are related to the mechanical properties of DNA, which makes it possible to adequately take into account various aspects of the DNA molecule functioning within the framework of a mechanical mathematical model of the angular movements of nitrogenous bases.

## 2. Mathematical Model

The mathematical model of the angular movements of nitrogenous bases is based on the analogy between a DNA molecule and a mechanical system consisting of two chains of interconnected pendulums. At the same time, nitrogenous bases correspond to the rotating pendulums, and the sugar-phosphate chains of the DNA molecule correspond to the elastic thread to which these pendulums are attached; the hydrogen bond of a pair of complementary nitrogenous bases corresponds to the elastic bond of the corresponding pair of pendulums [25,26,27].

Thus, the mathematical model of angular motions includes the following Newton equations:

(1) $$I_{1}^{i}\frac{d^{2}\varphi_{1}^{i}\left(t\right)}{dt^{2}}=K_{1}^{i}\left[\varphi_{1}^{i-1}\left(t\right)-2\varphi_{1}^{i}\left(t\right)+\varphi_{1}^{i+1}\left(t\right)\right]-\;-\delta^{i}\left(k_{12}^{i}R_{1}^{i}\left(R_{1}^{i}+R_{2}^{i}\right)\sin\varphi_{1}^{i}+k_{12}^{i}R_{1}^{i}R_{2}^{i}\sin\left(\varphi_{1}^{i}-\varphi_{2}^{i}\right)\right)+\;F_{1}^{i}\left(t\right),\;i=\bar{2,n-1},$$

(2) $$I_{1}^{1}\frac{d^{2}\varphi_{1}^{1}\left(t\right)}{dt^{2}}=K_{1}^{1}\left[\varphi_{1}^{2}\left(t\right)-\varphi_{1}^{1}\left(t\right)\right]-\;-\delta^{i}\left(k_{12}^{1}R_{1}^{1}\left(R_{1}^{1}+R_{2}^{1}\right)\sin\varphi_{1}^{1}+k_{12}^{1}R_{1}^{1}R_{2}^{1}\sin\left(\varphi_{1}^{1}-\varphi_{2}^{1}\right)\right)+F_{1}^{1}\left(t\right),$$

(3) $$I_{1}^{n}\frac{d^{2}\varphi_{1}^{n}\left(t\right)}{dt^{2}}=K_{1}^{n}\left[\varphi_{1}^{n-1}\left(t\right)-\varphi_{1}^{n}\left(t\right)\right]-\;-\delta^{i}\left(k_{12}^{n}R_{1}^{n}\left(R_{1}^{n}+R_{2}^{n}\right)\sin\varphi_{1}^{n}+k_{12}^{n}R_{1}^{n}R_{2}^{n}\sin\left(\varphi_{1}^{n}-\varphi_{2}^{n}\right)\right)+F_{1}^{n}\left(t\right),$$

(4) $$I_{2}^{i}\frac{d^{2}\varphi_{2}^{i}\left(t\right)}{dt^{2}}=K_{2}^{i}\left[\varphi_{2}^{i-1}\left(t\right)-2\varphi_{2}^{i}\left(t\right)+\varphi_{2}^{i+1}\left(t\right)\right]+\;+\delta^{i}\left(k_{12}^{i}R_{2}^{i}\left(R_{1}^{i}+R_{2}^{i}\right)\sin\varphi_{2}^{i}-k_{12}^{i}R_{1}^{i}R_{2}^{i}\sin\left(\varphi_{2}^{i}-\varphi_{1}^{i}\right)\right)+\;+F_{2}^{i}\left(t\right),\;i=\bar{2,n-1},$$

(5) $$I_{2}^{1}\frac{d^{2}\varphi_{2}^{1}\left(t\right)}{dt^{2}}=K_{2}^{1}\left[\varphi_{2}^{2}\left(t\right)-\varphi_{2}^{1}\left(t\right)\right]+\;\delta^{i}\left(k_{12}^{1}R_{2}^{1}\left(R_{1}^{1}+R_{2}^{1}\right)\sin\varphi_{2}^{1}1-k_{12}^{1}R_{1}^{1}R_{2}^{1}\sin\left(\varphi_{2}^{1}-\varphi_{1}^{1}\right)\right)+F_{2}^{1}\left(t\right),$$

(6) $$I_{2}^{n}\frac{d^{2}\varphi_{2}^{n}\left(t\right)}{dt^{2}}=K_{2}^{n}\left[\varphi_{2}^{n-1}\left(t\right)-\varphi_{2}^{n}\left(t\right)\right]+\;+\delta^{i}\left(k_{12}^{n}R_{2}^{n}\left(R_{1}^{n}+R_{2}^{n}\right)\sin\varphi_{2}^{n}-k_{12}^{n}R_{1}^{n}R_{2}^{n}\sin\left(\varphi_{2}^{n}-\varphi_{1}^{n}\right)\right)+F_{2}^{n}\left(t\right).$$

Here:

$\varphi_{j}^{i}\left(t\right)$—angular deviation of the *i*-pendulum of the *j*-chain, counted counterclockwise, at time *t*;

$I_{j}^{i}$—the moment of inertia of the *i*-pendulum of the *j*-chain;

$R_{j}^{i}$—distance from the center of mass of the *i*-pendulum of the *j*-chain to the thread;

$K_{j}^{i}$—constant characterizing the torque of the *i*-section of the *j*-thread;

$k_{12}^{i}$—constant characterizing the elastic properties of the connection of the *i*-pair of pendulums;

$F_{j}^{i}\left(t\right)$—external influence on the i-pendulum of the *j*-chain at time *t*;

n—the number of pairs of pendulums in the system.

In Equations (1) and (2), the first term to the right of the equal sign describes the force on the *i*-th pendulum from the side of the elastic thread, the second term—from the side of the paired pendulum, the third term—the external force. The magnitude of the external influence is taken equal to $F_{j}^{i}\left(t\right)=-\beta_{j}^{i}\frac{d\varphi_{j}^{i}}{dt}\left(t\right)+M^{i}\left(t\right)$, where the term $-\beta_{j}^{i}\frac{d\varphi_{j}^{i}}{dt}\left(t\right)$ models the effects of dissipation caused by the interaction with the liquid media surrounding the DNA molecule, the term $M^{i}\left(t\right)$—torque.

Equations (1)–(6) allow modeling the hydrogen bond in the *i*-th pair ($\delta^{i}=1$) and breaking this bond ($\delta^{i}=0$). We assume that a break occurs in the *i*-th pair of bases if the potential binding energy in this pair exceeds a certain critical value *E_H_*: the bond is restored if its potential energy is less than *E_H_* and −π < $\varphi_{1}^{i}$< π, 0 < $\varphi_{2}^{i}$ < 2π.

We add the initial conditions to Equations (1)–(6):

(7) $$\varphi_{1}^{i}\left(0\right)=\varphi_{1,0}^{i},\;\frac{d\varphi_{1}^{i}}{dt}\left(0\right)=\varphi_{1,1}^{i},\;$$

(8) $$\varphi_{2}^{i}\left(0\right)=\varphi_{2,0}^{i},\;\frac{d\varphi_{2}^{i}}{dt}\left(0\right)=\varphi_{2,1}^{i},\;i=\bar{1,n}.\;$$

For definiteness, we assume that in the initial conditions (7) and (8) are:

$$\varphi_{1,0}^{i}=\varphi_{1,1}^{i}=\varphi_{2,1}^{i}=0,\;\varphi_{2,0}^{i}=\pi ,\;i=\bar{1,n}.\;$$

## 3. Solution of a Model Problem

**Figure 1:**
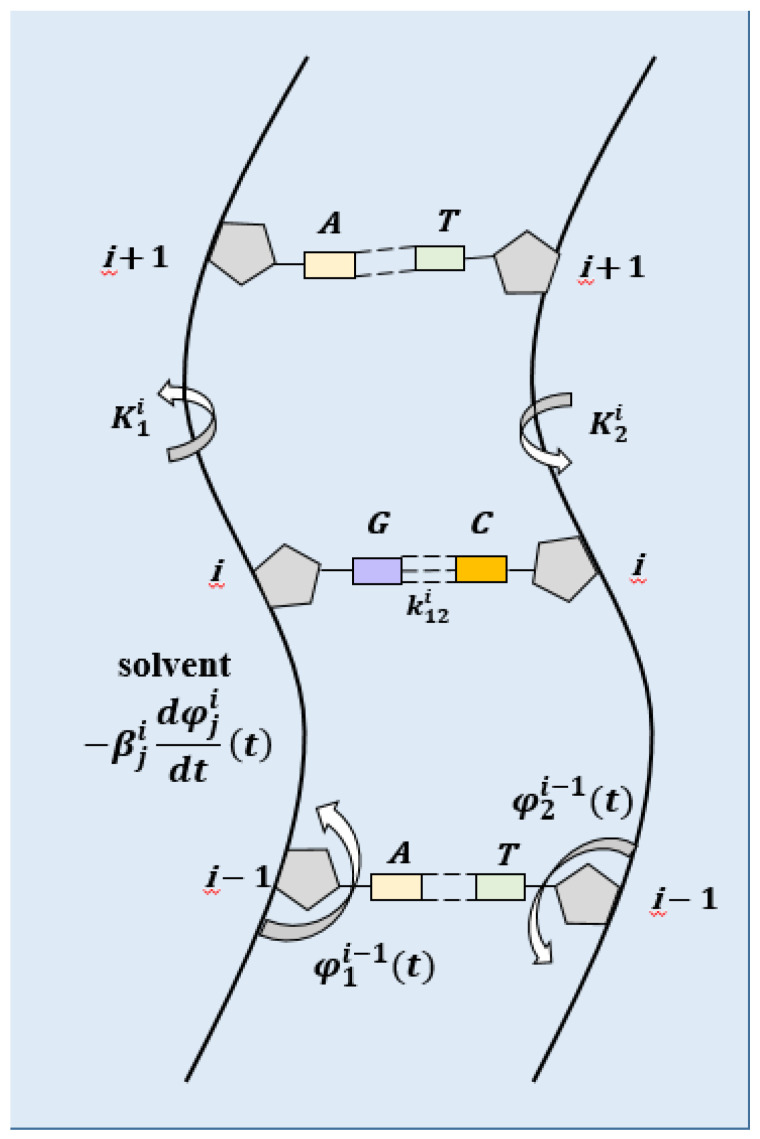
Graphic illustration of the model.

The process of formation and the dynamics of OS zones in the DNA molecule were studied on the basis of the numerical solution of problems (1)–(8). A graphic illustration of the model is shown in Figure 1.

The studies were carried out using the interferon alpha 17 gene code. For this gene, *n* = 980, the values of the coefficients of Equations (1)–(6) are given in Table 1, the energy value required to break one hydrogen bond is equal to *E_H_* = 34.774 pN·nm ≈ 5 kcal/mol (data taken from [25]).

## 4. Constant Torque

Let $M^{i}\left(t\right)$ = $M_{0}\cdot 1\;\mathrm{pN}\cdot \mathrm{nm}$, *i* = $\bar{1,\;n}$, *T*_0_ = 10^−10^ s. Calculations on the interval [0, 3*T*_0_] for $M_{0}$ with a step equal to 0.001 show that OS zones arise at *M*_0_ ≥ 13.173.

**Figure 2:**
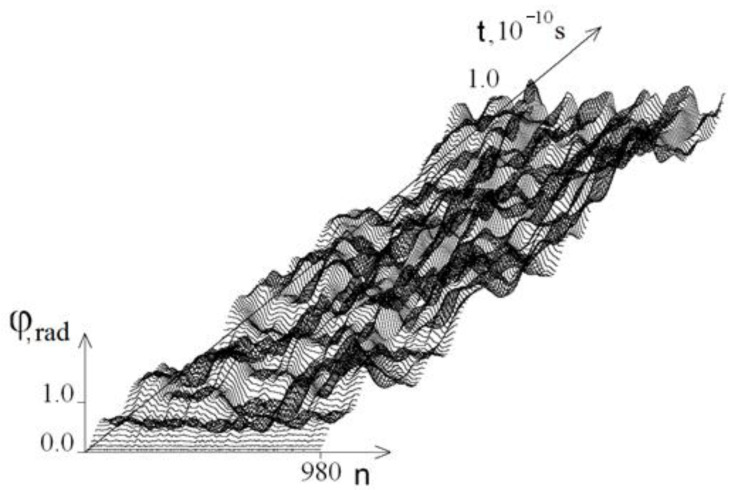
Graphs of angular deviations of the first strand of the DNA molecule at *M*_0_ = 13.172 on the time interval [0, *T*_0_].

**Figure 3:**
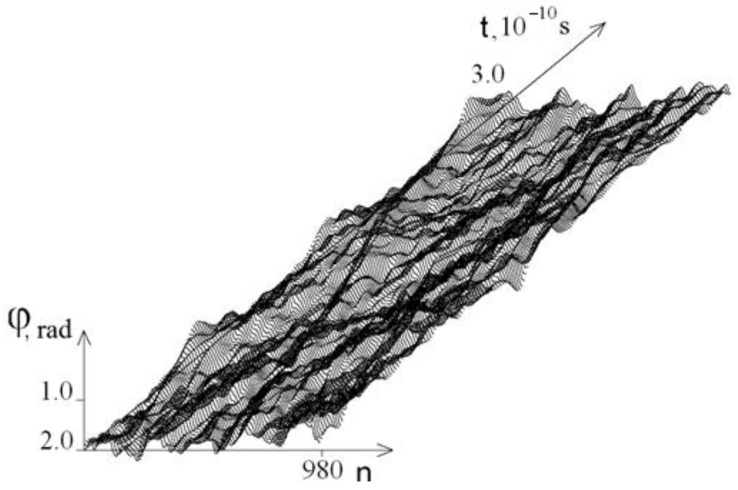
Graphs of angular deviations of the first strand of the DNA molecule at *M*_0_ = 13.172 on the time interval [2*T*_0_, 3*T*_0_].

Figure 2 and Figure 3 show the graphs of the base pairs’ angular deviations of the DNA molecule first strand at *M*_0_ = 13.172 on the intervals [0, *T*_0_] and [2*T*_0_, 3*T*_0_], respectively.

**Figure 4:**
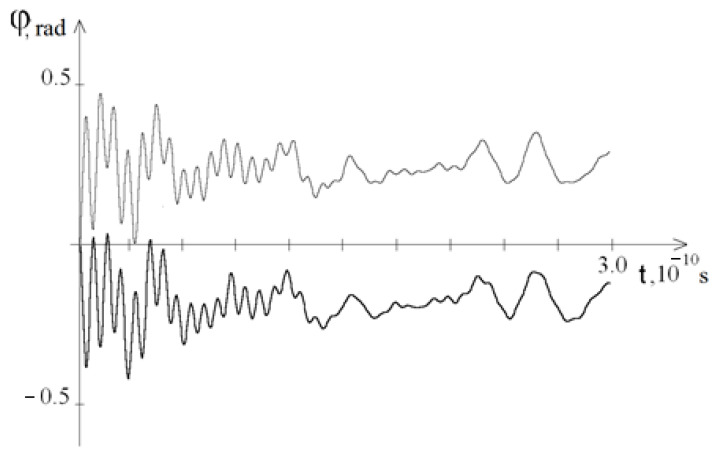
Graphs of the average angular deviations of the first (thin line) and second (thick line) strands of DNA nitrogenous bases at *M*_0_ = 13.172 over the time interval [0, 3*T*_0_].

Figure 4 shows graphs of the average angular deviations of the first (thin line) and second (thick line) strands of the DNA molecule (at *M*_0_ = 13.172), i.e.,

$$\varphi_{j}\left(t\right)=n^{-1}\sum_{i=1}^{n}\varphi_{j}^{i}\left(t\right),\;j=1,\;2$$

Figure 2 and Figure 3 show that at *t* ≥ *T*_0,_ the amplitude of the angular deviations of the DNA molecule chains decreases, and Figure 4 shows that the difference between the average angular deviations φ_1_ (*t*) − φ_2_ (*t*) also decreases; this explains why at *t* > *T*_0_, no new OS zones are formed and the unwinding of the DNA molecule process finished. Therefore, further calculations were carried out on the interval [0, *T*_0_].

**Figure 5:**
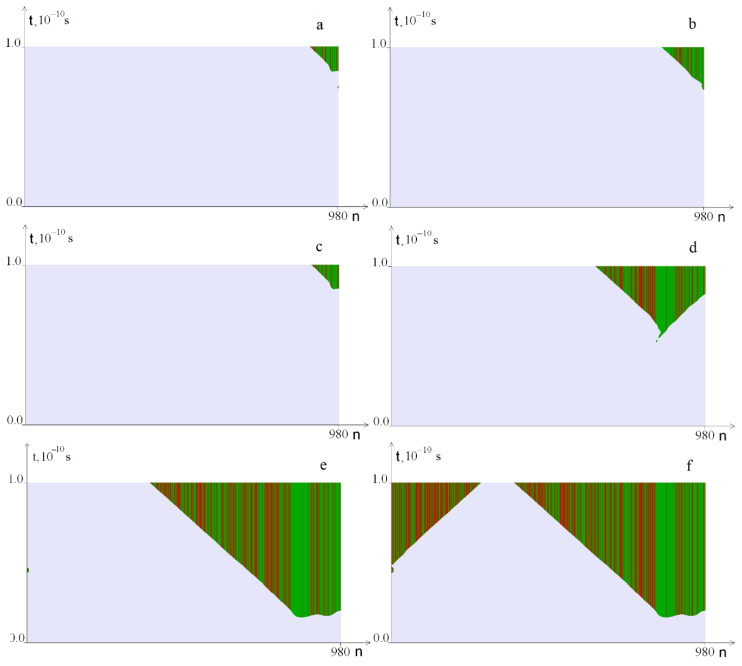
OS regions under different impact values of the torque on a DNA molecule *M*_0_: (**a**) at *M*_0_ = 13.173; (**b**)—at *M*_0_ = 13.185; (**c**)—at *M*_0_ = 13.210; (**d**)—at *M*_0_ = 13.673; (**e**)—at *M*_0_ = 15.794; (**f**)—at *M*_0_ = 15.795. Green indicates OS in *A–T* pairs, red, in *G–C* pairs.

The results of calculations for different *M*_0_ values from the range 13.173 ÷ 16 are shown in Figure 5; OS regions in pairs *A–T* are highlighted in green, and *G–C,* in red.

Figure 5 shows that with an increase in the torque value *M*_0_, the OS zone increases. Figure 5c–f show that as *M*_0_ increases, OS nucleation starts in the zone saturated with *A–T* pairs. With a significant increase in the torsion moment (*M*_0_ ≥ 15.795), OS zones begin to form near the left edge of the gen (Figure 5f).

**Figure 6:**
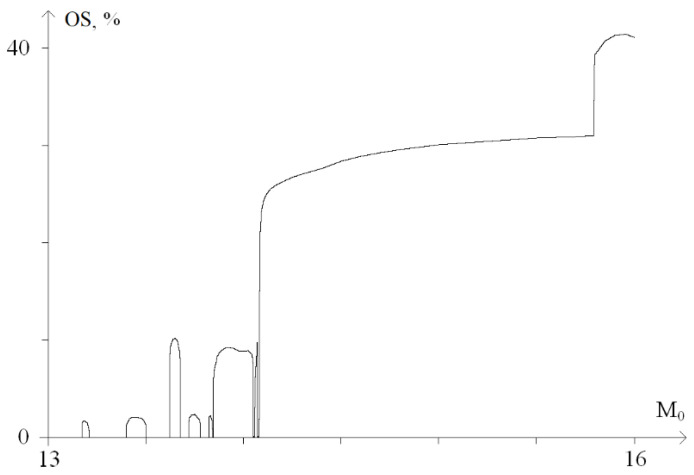
Graph of the probability (in percent) of OS occurrence under the influence of a constant torsion moment.

For a graphical representation of the dynamics of these processes, a graph of the probability (in percent) of the occurrence of OS for *M*_0_ from the range of 13 ÷ 16, was plotted (Figure 6).

Figure 6 shows that with a torque increase in the range of 13 to 14.1, nonlinear dependence of the OS occurrence probability is observed. Moreover, at *M*_0_ > 14.1, there is a sharp increase in the probability of OS occurrence up to 27%. Further, at 14.1 < *M*_0_ < 15.6, there is a gradual increase in the probability of OS occurrence from 27% to 32% and a sharp increase at *M*_0_ > 15.6, which corresponds to Figure 5e,f.

## 5. Spatially Localized Constant Torque

It is known that under real conditions, the action of the torque created by RNA occurs not on the entire gene, but on its promoter part. Therefore, below are the calculations for a localized torque acting on different parts of the interferon alpha gene.

Let $M^{i}\left(t\right)$ = $M_{0}^{i}\cdot 1\;\mathrm{pN}\cdot \mathrm{nm}$ *i* = $\bar{1,\;n}$, *t* ∈ [0, *T*_0_], and $M_{0}^{i}\left(t\right)=$ $M_{0}$ at 1 ≤ *i*_1_ ≤ *i* ≤ *i*_2_ ≤ *n,* $M_{0}^{i}\;$= 0 for other *i* values. Such a torsion action will be called spatially localized on the segment [*i*_1_, *i*_2_].

Figure 7, Figure 8, Figure 9, Figure 10, Figure 11, Figure 12, Figure 13 and Figure 14 show the results of calculations obtained for the different values of the torque, localized on segments with a length of 100 base pairs: [1, 100], [101, 200], [781, 880] and [881, 980].

**Figure 7:**
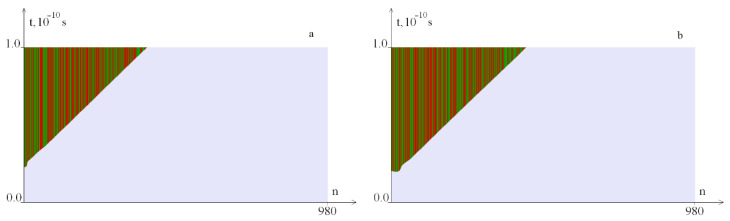
OS regions under the local torque influence on 1–100 pairs of nitrogenous bases with different *M*_0_ values: (**a**)—at *M*_0_ = 16.8; (**b**)—at *M*_0_ = 16.9; green indicates OS in *A–T* pairs, red, in *G–C* pairs.

Figure 7 shows OS regions under the torque localized in the segment [1, 100]. It can be seen that with an increase in the *M*_0_ value, the OS zones increase. Note, that the zone from 1 to 100 base pairs of the nitrogenous base pairs of the interferon alpha gene contains a large number of *G–C* pairs.

**Figure 8:**
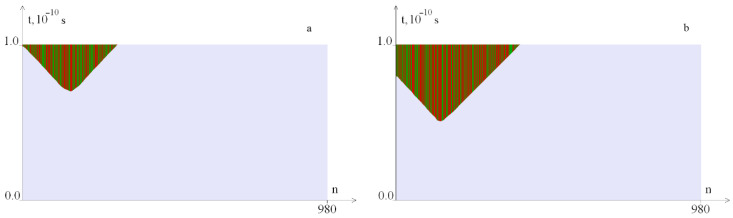
OS sections under the localized torque action (*M*_0_: (**a**)—at *M*_0_ = 22.2, (**b**)—at *M*_0_ = 22.3) on bases 101–200. Green indicates OS in *A–T* pairs, red, in *G–C* pairs.

Figure 8 shows OS regions under the localized torque action on the segment [101, 200]. It can be seen that with an increase of the *M*_0_ value, the OS zones also increase. Note that for the occurrence of OS zones, a larger *M*_0_ value is required than when it is localized in the segment [1, 100]. This can be explained by the fact that a smaller amount of energy is required for the occurrence of OS when the torque effect is localized at the end of the gen (edge effect). 

**Figure 9:**
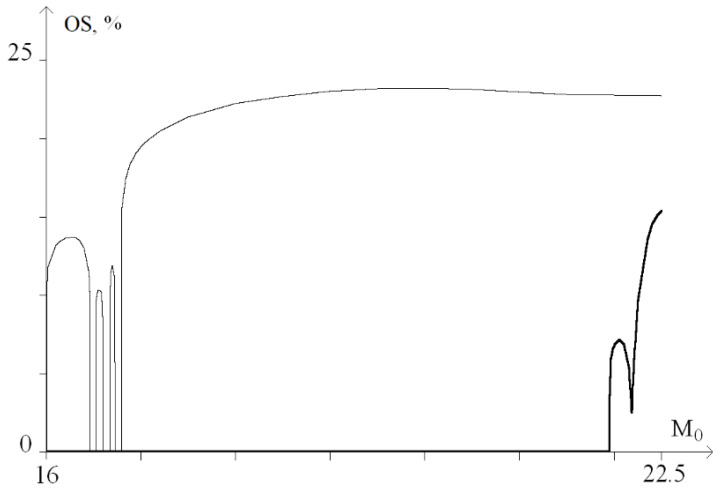
Graphs of the probability (in percent) of OS occurrence under the action of a torsion moment localized on the segment [1, 100] (thin line) and on the segment [101, 200] (thick line).

Figure 9 shows the graphs of the OS occurrence probability under the localized torque action on the segments [1, 100] and [101, 200].

**Figure 10:**
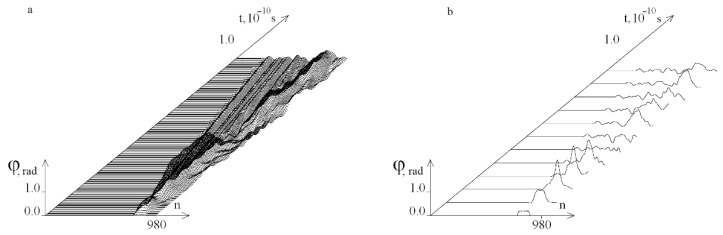
Graphs of the angular deviations of the first DNA molecule strand under the localized torque action on the interval [781, 880] at *M*_0_ = 15.045 on the time interval [0, *T*_0_]. (**a**) shows 200 graphs and (**b**) has 12 graphs.

Figure 10 shows graphs of the angular deviations of the first DNA molecule of the strand under the localized torque action on the segment [781, 880] at *M*_0_ = 15.045, at which OS does not yet occur.

**Figure 11:**
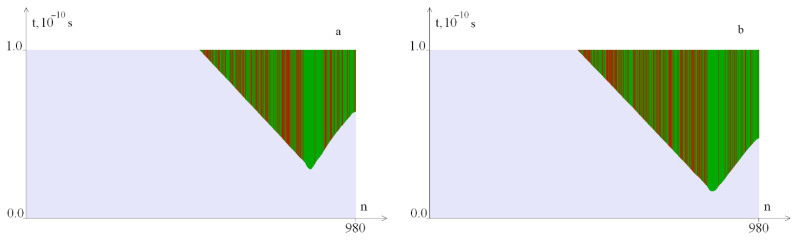
OS regions arising under the localized torque action on the segment [781, 880] ((**a**)—at *M*_0_ = 15.046, (**b**)—at *M*_0_ = 16). Green indicates OS in *A–T* pairs, red, in *G–C* pairs.

Figure 11 shows OS regions under the localized torque action on the segment [781, 880]. It can be seen, that the occurrence of OS when the torque is localized in the area rich with *A–T* pairs begins at lower values of the *M*_0_ opposite torque localization on the segments [1, 100] and [101, 200].

**Figure 12:**
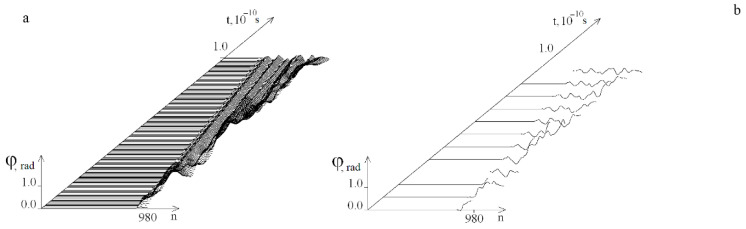
Graphs of the angular deviations of the DNA molecule first chain under the localized torque action on the interval [881, 980] at *M*_0_ = 14.032 on the time interval [0, *T*_0_]. (**a**) shows 200 graphs and (**b**) has 12 graphs.

Figure 12 shows graphs of the angular deviations of the first strand of the DNA molecule under the localized torque action on the segment [881, 980] at *M*_0_ = 14.032, at which OS does not yet occur.

**Figure 13:**
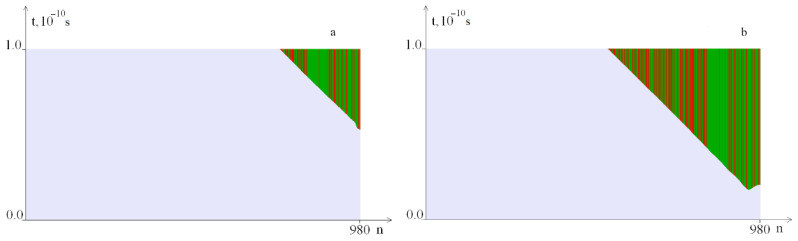
OS regions arising under the localized torque action on the segment [881, 980] for different *M*_0_ values: (**a**)—at *M*_0_ = 14.033, (**b**)—at *M*_0_ = 16. Green color indicates OS in *A–T* pairs, red, in *G–C* pairs.

Figure 13 shows OS regions under the localized torque action on the interval [881, 980]. It can be seen that the occurrence of OS, in this case, occurs at even lower values of the torque than in the case of localization on the region [781, 880], which can be explained by the edge effect.

**Figure 14:**
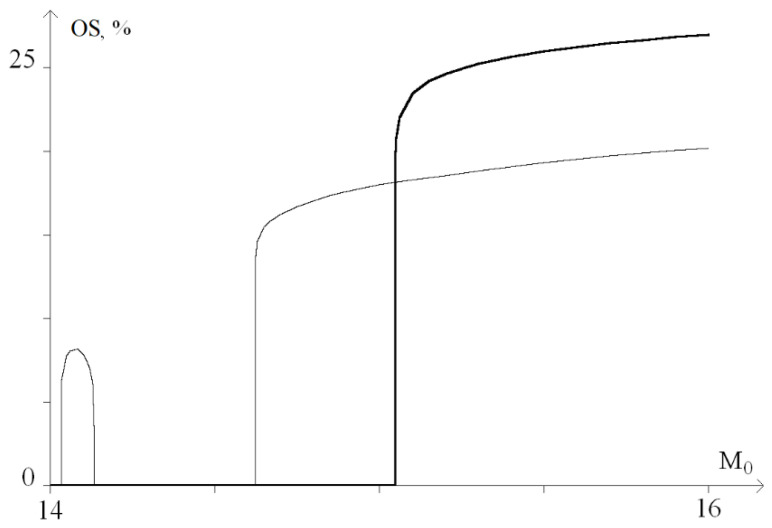
Graphs of the probability (in percent) of OS occurrence under the localized torque action on the segment [781, 880] (thin line) and the segment [881, 980] (thick line).

Figure 14 shows the graphs of the OS occurrence probability under the localized torque action on the segments [781, 880] and [881, 980].

**Figure 15:**
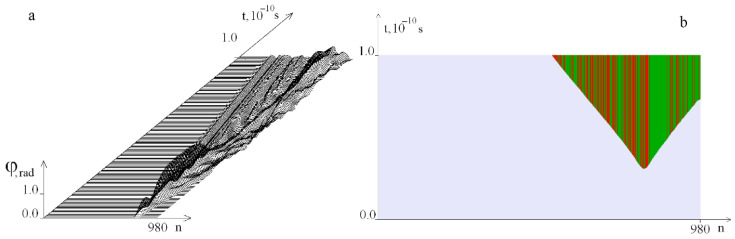
Influence of localized torque on a segment [781, 830]. (**a**)—Graph of the angular deviations of the first strand of the DNA molecule on the time interval [0, *T*_0_] at *M*_0_ = 21.12. (**b**)—OS regions at *M*_0_ = 21.121, green color indicates OS in *A–T* pairs, red, in *G–C* pairs.

**Figure 16:**
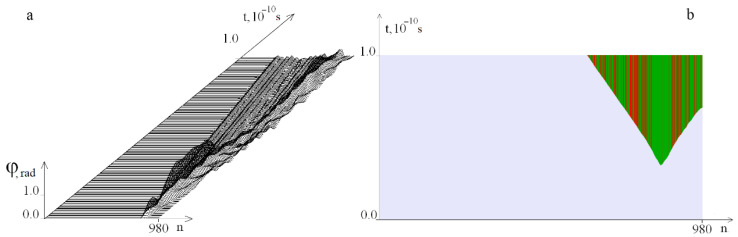
Influence of localized torque on a segment [831, 880]. (**a**)—Graph of the angular deviations of the first strand of the DNA molecule on the time interval [0, *T*_0_] at *M*_0_ = 18.232. (**b**)—OS regions at *M*_0_ = 18.233, green color indicates OS in *A–T* pairs, red, in *G–C* pairs.

**Figure 17:**
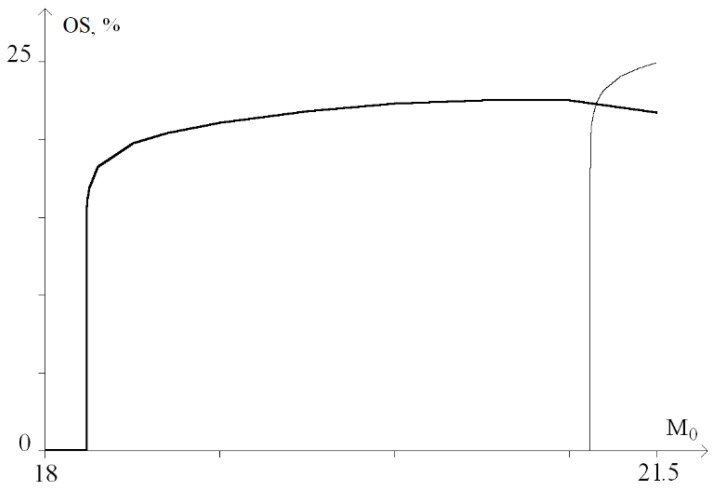
Graphs of the probability (in percent) of OS occurrence under the localized torque action on the segment [781, 830] (thin line) and the segment [831, 880] (thick line).

Figure 15, Figure 16 and Figure 17 show the results of calculations obtained for the localized torque action on segments 50 base pairs long: [781, 830] and [831, 880]. Graphs of angular deviations are shown at the highest *M*_0_ value, which does not lead to OS.

Figure 17 shows the graphs of the OS occurrence probability under the localized torque action on the segments [781, 830] and [831, 880]. The differences in the graphs are explained by the fact that section [831, 880] contains many more *A–T* pairs. In addition, the reduction of localized torque segments from 100 to 50 pairs led to an increase in the *M*_0_ values required for the occurrence of OS zones.

## 6. Spatio-Temporal Localized Torsion Moment

Let $M^{i}\left(t\right)$ = $M_{0}^{i}\left(t\right)\cdot 1\;\mathrm{pN}\cdot \mathrm{nm}$, *i* = $\bar{1,\;n}$, *t* ∈ [0, *T*_0_], and $M_{0}^{i}\left(t\right)=$ $M_{0}$ at 1 ≤ *i*_1_ ≤ *i* ≤ *i*_2_ ≤ *n,* 0 < *t*_1_ ≤ *t* ≤ *t*_2_ ≤ *T*_0_ and $M_{0}^{i}\left(t\right)$ = 0 for other *i* and *t* values. Such a torsion effect will be called localized on a spatial segment [*i*_1_, *i*_2_] and time interval [*t*_1_, *t*_2_].

**Figure 18:**
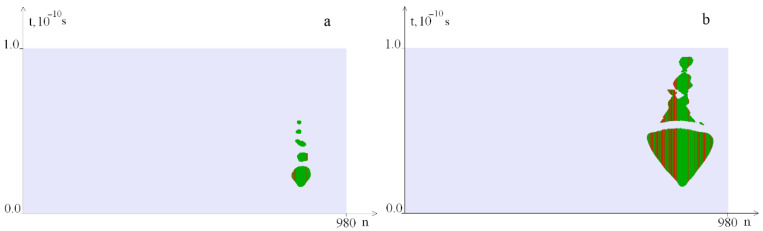
OS sections arising under the localized torque action on the interval [781, 880] at *M*_0_ = 16. (**a**)—on the time interval [0, 0.2*T*_0_] and (**b**)—on the time interval [0, 0.25*T*_0_]. Green color indicates OS in *A–T* pairs, red, in *G–C* pairs.

Figure 18 shows OS regions that arise under the localized torque action on the spatial segment [781, 880] with different temporal localization. It can be seen that when the torsion action stops, the OS zones are closed, and an increase in the time localization interval leads to an increase in the OS zones.

## 7. Conclusions

In the article [19], it was shown that the torque value for RNAp *Escherichia coli* is 11 ± 4 pN·nm. Our simulation results showed that the torque values required for the occurrence of OS zones depend on the localization and, in some cases, are close to those obtained in the experimental work values [19].

It follows from the obtained results that the probability of OS zones formation depends on the content of *A–T* pairs in the area of torque localization, the size of this area and the size of the time interval influence. It should be noted that the torque spatial localization near the edge of the gen leads to an increase in the probability of the occurrence of OS zones (edge effect), and also the OS zones can close when the torsion action stops.

In addition, it was noted that the torsion effect leads to the genesis of rotational movements of nitrogenous bases. This effect is nonlinear, and an increase in the amplitude of the torsion action does not automatically increase the amplitude of rotational movements and the potential energy of hydrogen bonds and, as a result, increases the OS regions. This explains the non-monotonicity of the OS occurrence probability graphs (Figure 6, Figure 9, Figure 14 and Figure 17).

## Figures and Tables

**Table 1:** Coefficients of Equations (1)–(6).

Type of Base	A	T	G	C
$I\cdot 10^{-44},\;\mathrm{kg}\cdot m^{2}$	7.61	4.86	8.22	4.11
R, Å	5.80	4.80	5.70	4.70
$K\cdot 10^{-18}$, N·m	2.35	1.61	2.27	1.54
$k_{12}^{H}\cdot 10^{-2},\;N/m$	6.20	6.20	9.60	9.60
$\beta \cdot 10^{-34},\;N\cdot m\cdot s$	4.25	2.91	4.10	2.79

## Data Availability

Not applicable.
